# Diffusion in a disk with inclusion: Evaluating Green’s functions

**DOI:** 10.1371/journal.pone.0265935

**Published:** 2022-04-14

**Authors:** Remus Stana, Grant Lythe

**Affiliations:** Department of Applied Mathematics, University of Leeds, Leeds, United Kingdom; Utrecht University, NETHERLANDS

## Abstract

We give exact Green’s functions in two space dimensions. We work in a scaled domain that is a circle of unit radius with a smaller circular “inclusion”, of radius *a*, removed, without restriction on the size or position of the inclusion. We consider the two cases where one of the two boundaries is absorbing and the other is reflecting. Given a particle with diffusivity *D*, in a circle with radius *R*, the mean time to reach the absorbing boundary is a function of the initial condition, given by the integral of Green’s function over the domain. We scale to a circle of unit radius, then transform to bipolar coordinates. We show the equivalence of two different series expansions, and obtain closed expressions that are not series expansions.

## Introduction

Brownian motion is a common model of microscopic behaviour, such as that of intracellular molecules [1–4]. Depending on whether the mathematical interest is in statistics of many particles, or in single-particle properties such as mean hitting times, the diffusion, Laplace, or Poisson equation may need to be solved [5–8]. Absorption or reflection at surfaces is expressed in terms of boundary conditions. Green’s function is the key to analytical solutions because it takes the shape of the domain and the boundary conditions into account. Quantities such as mean hitting times are obtained from it by standard integration, for any initial distribution [9–14]. It is also possible to model a surface with both absorbing and reflecting parts using Robin boundary conditions [15–17].

The domain we consider here is a circle of unit radius with a smaller circular “inclusion”, of radius *a*, removed. The centre of the inclusion is displaced from the centre of the circle of unit radius by *c*, with 0 ≤ *c* ≤ 1 − *a*. We consider the two cases where one circle is an absorbing boundary, the other is reflecting (reflecting inclusion inside a circular domain with absorbing boundary, and *vice versa*). In [14], the circle of unit radius was referred to as the cellular surface and the inclusion as the cell’s nucleus. In two and three dimensions, Condamin *et al*. [18], constructed approximate Green’s functions. Asymptotic and numerical methods can be used when there are multiple targets of different shapes in a two-dimensional region [19–22]. They are accurate when the targets are not too large, not too close to each other and not too close to the cellular surface. The functions given here are, however, exact without restriction on the size or position of the inclusion.

**Fig 1:**
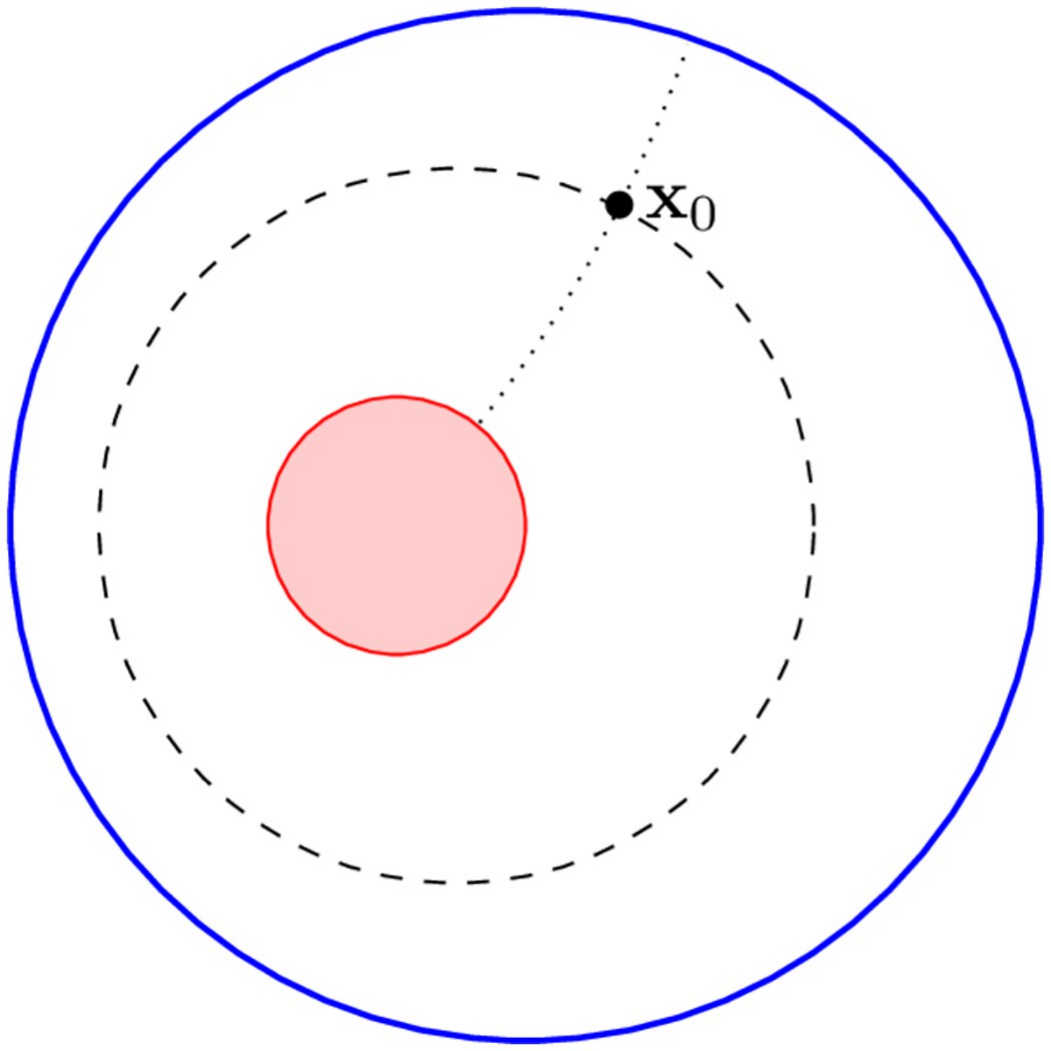
The domain is the interior of the unit circle (blue) with a circular inclusion (red). The initial position of a diffusing particle, **x**_0_, has bipolar coordinates *τ*_0_, *σ*_0_. The dashed circle is the set of points with *τ* = *τ*_0_. The dotted arc is part of the set of points with *σ* = *σ*_0_. *G*_1_(**x**_0_, **x**) is the solution of (4), constrained to be zero on the unit circle and to have normal derivative on the inclusion. *G*_2_(**x**_0_, **x**) is the solution of (4), constrained to be zero on the inclusion and to have normal derivative on the unit circle.

We use bipolar coordinates, *τ* and *σ* (Fig 1); the circle of unit radius has *τ* = *τ*_2_ and the boundary of the inclusion has *τ* = *τ*_1_, where

$$\begin{array}{c} \tau_{1}=\text{log}\;(d/a+\sqrt{1+{\left(d/a\right)}^{2}}),\;\tau_{2}=\text{log}\;(d+\sqrt{1+d^{2}}) \end{array}$$ (1)

and

$$\begin{array}{c} d=\frac{1}{2c}\sqrt{{\left(1+a^{2}-c^{2}\right)}^{2}-4a^{2}}. \end{array}$$ (2)

We calculate Green’s functions on the rectangular domain in bipolar coordinates *τ*_2_ ≤ *τ* ≤ *τ*_1_, 0 ≤ *σ* ≤ 2*π*; mean exit times are calculated by integrating over the coordinates (*τ*, *σ*) [14].

The transformation from Cartesian to bipolar coordinates, (*x*, *y*) to (*τ*, *σ*), is a type of conformal transformation employed, for example, to express the electric potential between two parallel cylinders [5, 23]. Another example of a conformal transformation is the bilinear function

$$f\left(z\right)=\frac{z+\alpha }{z+\beta },$$

where *z* = *x* + i*y*. Circles are mapped to circles and, with suitable choices of *α* and *β*, two nonconcentric circles can be mapped to two concentric ones. When one is the unit circle, *αβ* = 1 [24]. The bilinear transformation has been used to obtain solutions of Laplace’s equation with absorbing boundaries on both circles [25, 26]. We may construct Green’s functions in nonconcentric domains from those in concentric domains, $\tilde{G}$, which are also series expansions [27], as $\tilde{G}(f^{-1}(z_{0}),f^{-1}(z))$. However, the integrals needed to calculate mean exit times have only been performed using bipolar coordinates [14].

Green’s function *G*(**x**_0_, **x**) is a symmetric function of two positions **x**_0_ and **x**, where **x**_0_ is taken to be the position of a point charge or the initial condition of a diffusing particle. As a result, *G*(**x**_0_, **x**) is proportional to − log |**x** − **x**_0_| as **x** → **x**_0_. Writing it as a sum of singular and regular parts, and expressing both in bipolar coordinates [5, 28], Heyda was able to find a series expression for Green’s function with absorbing boundaries. A different approach to the same problem [29], because the transformed domain is rectangular, is to expand Green’s function in trigonometric eigenfunctions. The resulting series solution can be summed to yield an explicit expression involving Jacobi Theta functions [29]. Heyda’s method has recently been applied to the problem where one circular boundary is absorbing and the other is reflecting [14]. Explicit exact solutions are useful, even when they are series, because they can be integrated to yield mean transport times, or expanded in small parameters to yield simple expressions, depending on the geometry and dynamics of the context of diffusion in confined geometries [8, 30–35].

Given a particle with diffusivity *D*, in a circle with radius *R*, the mean time to reach an absorbing boundary is a function of the initial condition, given by the integral of Green’s function over the domain. Given *R*, we firstly scale to a circle of unit radius, then transform to bipolar coordinates. With the Jacobian factor of the transformation, *d*^2^/(cosh *τ* − cos *σ*)^2^, the integral is written as

$$\begin{array}{c} T\left({\text{x}}_{0}\right)=\frac{R^{2}}{D}\int_{\tau_{2}}^{\tau_{1}}\int_{0}^{2\pi }\frac{G\left({\text{x}}_{0},\text{x}\right)d^{2}}{{\left(\text{cosh}\;\tau -\text{cos}\;\sigma \right)}^{2}}d\sigma d\tau . \end{array}$$ (3)

Green’s function satisfies

$$\begin{array}{c} \Delta_{\text{x}}G\left({\text{x}}_{0},\text{x}\right)=-\delta \left(\text{x}-{\text{x}}_{0}\right)\;\text{x}\in C, \end{array}$$ (4)

with conditions on the boundaries of *C*. We consider two cases.

- *G*_1_(**x**_0_, **x**) is the solution of (4), constrained to be zero when *τ* = *τ*_2_ and to have normal derivative zero when *τ* = *τ*_1_. The corresponding time obtained from (3) is the mean time for a diffusing particle to reach the boundary of the circle of radius *R*, when the boundary of the inclusion is reflecting.
- *G*_2_(**x**_0_, **x**) is the solution of (4), constrained to be zero when *τ* = *τ*_1_ and to have normal derivative zero when *τ* = *τ*_2_. The corresponding time obtained from (3) is the mean time for a diffusing particle to reach the inclusion, when the boundary of the circle of radius *R* is reflecting.

In terms of (*τ*, *σ*), the bipolar coordinate representation of **x**, and (*τ*_0_, *σ*_0_), the representation of **x**_0_, we define *τ*_A_ = min(*τ*, *τ*_0_) − *τ*_2_, *τ*_*B*_ = *τ*_1_ − max(*τ*, *τ*_0_) and *θ* = |*σ* − *σ*_0_| − *π*, where −*π* < *θ* ≤ *π*.

## Equivalence of series

Two different series expressions exist for *G*_1_ and *G*_2_. Based on the form used by Heyda [36], we can write [14]

$$2\pi G_{1}({\text{x}}_{0},\text{x})=\tau_{A}+\sum_{m=1}^{\infty }\frac{2}{m}\frac{\text{sinh}\;m\tau_{A}\;\text{cosh}\;m\tau_{B}}{\text{cosh}\;m(\tau_{1}-\tau_{2})}\text{cos}\;m\theta$$ (5)

and

$$2\pi G_{2}({\text{x}}_{0},\text{x})=\tau_{B}+\sum_{m=1}^{\infty }\frac{2}{m}\frac{\text{sinh}\;m\tau_{B}\;\text{cosh}\;m\tau_{A}}{\text{cosh}\;m(\tau_{1}-\tau_{2})}\text{cos}\;m\theta .$$ (6)

A different summation, developed by Liemert [29], may be modified to the case of one absorbing and one reflecting boundary to yield

$$2\pi G_{1}({\text{x}}_{0},\text{x})=\sum_{n=0}^{\infty }\frac{4}{2n+1}\frac{\text{sin}\;(\lambda_{n}\left(\tau -\tau_{2}\right))\;\text{sin}\;(\lambda_{n}\left(\tau_{0}-\tau_{2}\right))}{\text{sinh}\;\lambda_{n}\pi }\text{cosh}\;\lambda_{n}\theta ,$$ (7)

and

$$2\pi G_{2}({\text{x}}_{0},\text{x})=\sum_{n=0}^{\infty }\frac{4}{2n+1}\frac{\text{sin}\;(\lambda_{n}\left(\tau_{1}-\tau \right))\;\text{sin}\;(\lambda_{n}\left(\tau_{1}-\tau_{0}\right))}{\text{sinh}\;\lambda_{n}\pi }\text{cosh}\;\lambda_{n}\theta ,$$ (8)

where

$$\lambda_{n}=\frac{(2n+1)\pi }{2(\tau_{1}-\tau_{2})}.$$ (9)

Our first aim is to demonstrate that these two expressions, superficially very different, are equivalent. To do so, we seek to write (5) in the form

$$2\pi G_{1}({\text{x}}_{0},\text{x})=\sum_{l=0}^{\infty }A_{l}\;\text{sin}(\lambda_{l}\left(\tau -\tau_{2}\right)),$$

where

$$A_{l}=\frac{4\pi }{\tau_{1}-\tau_{2}}\int_{\tau_{2}}^{\tau_{1}}G_{1}({\text{x}}_{0},\text{x})\;\text{sin}\;(\lambda_{l}\left(\tau -\tau_{2}\right))d\tau .$$

Thus

$$A_{l}=\frac{8}{\pi }\frac{\lambda_{l}}{2l+1}(\frac{1}{2\lambda_{l}^{2}}+\sum_{m=1}^{\infty }\frac{\text{cos}\;m\theta }{\lambda_{l}^{2}+m^{2}})\text{sin}\;\lambda_{l}\left(\tau_{0}-\tau_{2}\right).$$

Using

$$\begin{array}{c} \frac{\text{cosh}\;\lambda_{m}\theta }{\text{sinh}\;\lambda_{m}\pi }=\frac{2\lambda_{m}}{\pi }(\frac{1}{2\lambda_{m}^{2}}+\sum_{n=1}^{\infty }\frac{\text{cos}\;n\theta }{\lambda_{m}^{2}+n^{2}}), \end{array}$$

we find

$$A_{l}=\frac{4}{2l+1}\frac{\text{cosh}\;\lambda_{l}\theta }{\text{sinh}\;\lambda_{l}\pi }\;\text{sin}\;\lambda_{l}\left(\tau_{0}-\tau_{2}\right),$$

which is consistent with (7). Similarly, (6) is equivalent to (8).

## Evaluation without series

To obtain closed expressions that are not series expansions, we rearrange the summand in (7), using [29]

$$\begin{array}{c} 4\;\text{sin}\;(\lambda_{n}\left(\tau -\tau_{2}\right))\;\text{sin}\;(\lambda_{n}\left(\tau_{0}-\tau_{2}\right))\;\text{cosh}\;\lambda_{n}\theta \\ \;=ℜ(e^{i(2n+1)\beta }+e^{-i(2n+1)\beta }-e^{i(2n+1)\alpha }-e^{-i(2n+1)\alpha }), \end{array}$$

where ℜ(*z*) is the real part of *z*,

$$\begin{array}{c} \alpha =\frac{\pi }{2}\frac{\tau +\tau_{0}-2\tau_{2}+i\theta }{\tau_{1}-\tau_{2}},\;\beta =\frac{\pi }{2}\frac{\tau -\tau_{0}+i\theta }{\tau_{1}-\tau_{2}}, \end{array}$$ (10)

and

$$\begin{array}{c} \text{sinh}(\lambda_{n}\pi )=\frac{1-q^{2(2n+1)}}{2q^{2n+1}}\;\text{where}\;q=\text{exp}(-\frac{\pi^{2}}{2(\tau_{1}-\tau_{2})}). \end{array}$$ (11)

Then (7) is written [29]

$$\begin{array}{c} 2\pi G_{1}({\text{x}}_{0},\text{x})=\sum_{n=0}^{\infty }\sum_{k=1}^{\infty }\frac{q^{\left(2k-1)(2n+1\right)}}{2n+1}2ℜ(e^{i(2n+1)\beta }+e^{-i(2n+1)\beta }-e^{i(2n+1)\alpha }-e^{-i(2n+1)\alpha }). \end{array}$$

When |*z*| < 1,

$$\begin{array}{c} 2ℜ(\sum_{n=0}^{\infty }\frac{z^{2n+1}}{2n+1})=\text{log}|\frac{1+z}{1-z}|. \end{array}$$

Therefore

$$\begin{array}{cc} 2\pi G_{1}({\text{x}}_{0},\text{x}) & =\sum_{k=1}^{\infty }\text{log}|\frac{1+q^{2k-1}e^{i\beta }}{1-q^{2k-1}e^{i\beta }}\frac{1+q^{2k-1}e^{-i\beta }}{1-q^{2k-1}e^{-i\beta }}\frac{1-q^{2k-1}e^{i\alpha }}{1+q^{2k-1}e^{i\alpha }}\frac{1-q^{2k-1}e^{-i\alpha }}{1+q^{2k-1}e^{-i\alpha }}|\\ & =\sum_{k=1}^{\infty }\text{log}\frac{|1+2q^{2k-1}\text{cos}\beta +q^{4k-2}|}{|1-2q^{2k-1}\text{cos}\beta +q^{4k-2}|}\frac{|1-2q^{2k-1}\text{cos}\alpha +q^{4k-2}|}{|1+2q^{2k-1}\text{cos}\alpha +q^{4k-2}|}\\ & =\text{log}|\frac{\vartheta_{3}(\beta /2,q)\vartheta_{4}(\alpha /2,q)}{\vartheta_{4}(\beta /2,q)\vartheta_{3}(\alpha /2,q)}|, \end{array}$$ (12)

where *ϑ*_3_(*z*, *q*) and *ϑ*_4_(*z*, *q*) are Jacobi theta functions [29, 37]. Similarly,

$$\begin{array}{c} 2\pi G_{2}({\text{x}}_{0},\text{x})=\text{log}|\frac{\vartheta_{3}(\beta_{2}/2,q)\vartheta_{4}(\alpha_{2}/2,q)}{\vartheta_{4}(\beta_{2}/2,q)\vartheta_{3}(\alpha_{2}/2,q)}|, \end{array}$$ (13)

where

$$\begin{array}{c} \alpha_{2}=\frac{\pi }{2}\frac{2\tau_{1}-\tau -\tau_{0}+i\theta }{\tau_{1}-\tau_{2}}\;\text{and}\;\beta_{2}=\frac{\pi }{2}\frac{\tau_{0}-\tau +i\theta }{\tau_{1}-\tau_{2}}. \end{array}$$

**Fig 2:**
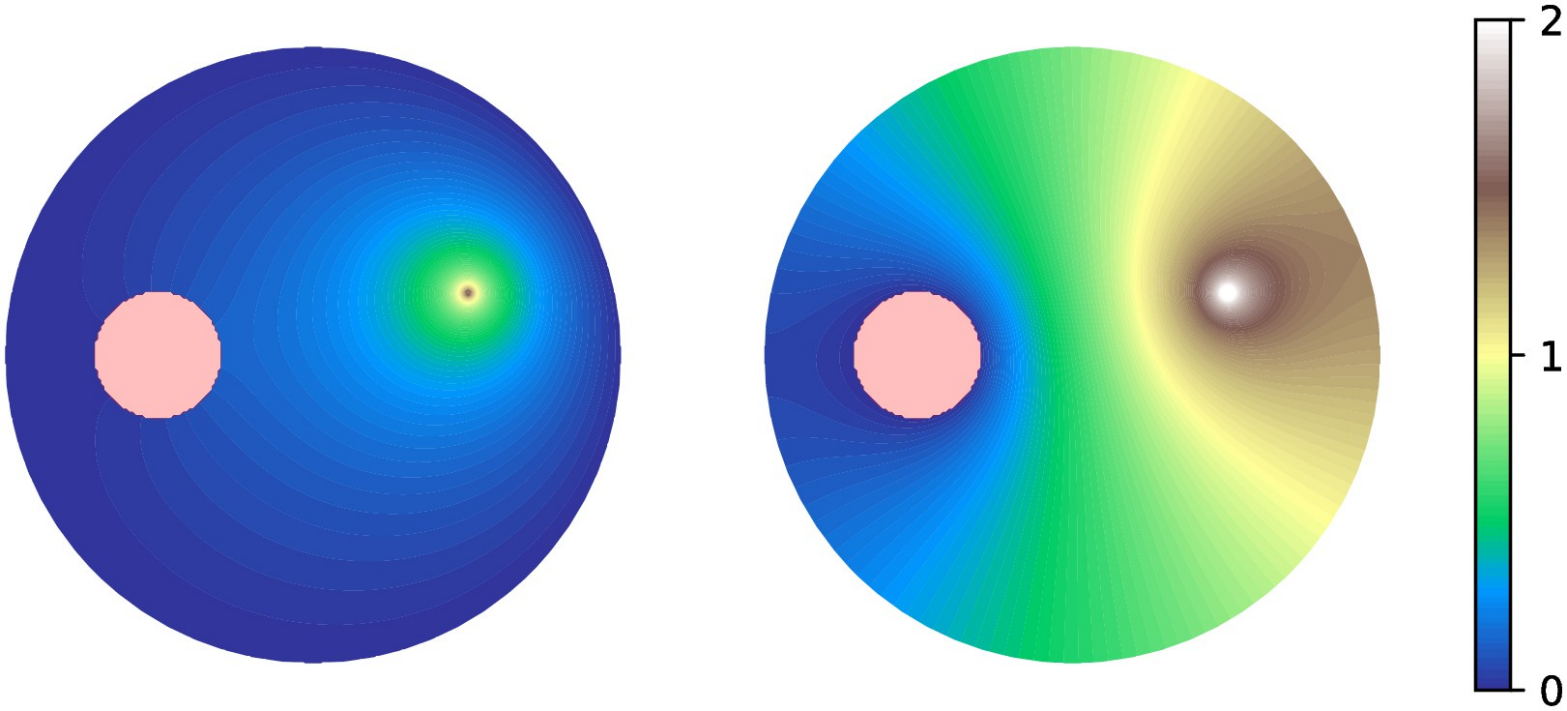
Exact Green’s functions. Left: *G*_1_(**x**_0_, **x**). The boundary of the unit circle is absorbing, the boundary of the inclusion is reflecting. Right: *G*_2_(**x**_0_, **x**). The boundary of the unit circle is reflecting, the boundary of the inclusion is absorbing. The initial position of the diffusing particle, **x**_0_, is displaced by (0.5, 0.2) from the center of the unit disk; the inclusion has radius 0.2 and is displaced by (−0.5, 0). The value of the function at **x** is the occupation density of the diffusing particle, until reaching the absorbing boundary. We use the closed expressions (12) and (13) that involve the Jacobi theta function. Python code is provided in the S1 Code.

In Fig 2, the dependence of *G*_1_(**x**_0_, **x**) and *G*_2_(**x**_0_, **x**) on **x** is shown, with **x**_0_ fixed, when *c* = 0.5 and *a* = 0.2. We use the closed expressions (12) and (13). Jacobi functions are available in many software packages; we give an example in the S1 Code.

**Fig 3:**
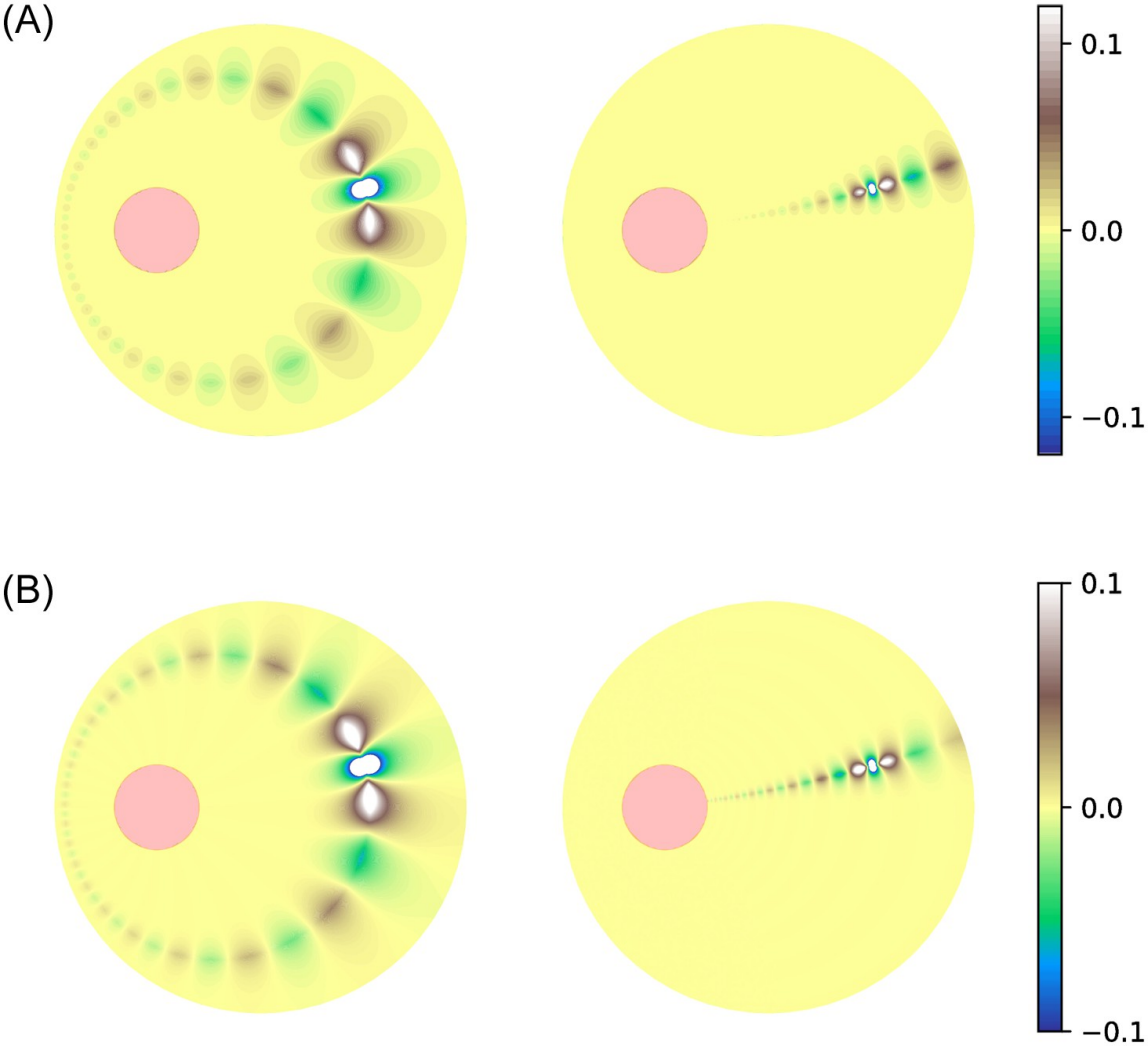
Dependence of truncation errors in Green’s functions on position x. The inclusion is the red disk, displaced by (−0.5, 0) with respect to the centre of the unit circle, and the initial position **x**_0_ of the diffusing particle, is displaced by (0.5, 0.2). Upper left: $G_{1}({\text{x}}_{0},\text{x})-G_{1}^{\text{H}}({\text{x}}_{0},\text{x},20)$. Upper right: $G_{1}({\text{x}}_{0},\text{x})-G_{1}^{\text{L}}({\text{x}}_{0},\text{x},20)$. Lower left: $G_{2}({\text{x}}_{0},\text{x})-G_{2}^{\text{H}}({\text{x}}_{0},\text{x},20)$. Lower right: $G_{2}({\text{x}}_{0},\text{x})-G_{2}^{\text{L}}({\text{x}}_{0},\text{x},20)$.

If truncated at a finite number of terms, the series expressions (5)–(8) are not exact. To consider the effect of only using a finite number of terms, we define

$$2\pi G_{1}^{H}({\text{x}}_{0},\text{x},n)=\tau_{A}+\sum_{m=1}^{n}\frac{2}{m}\frac{\text{sinh}\;m\tau_{A}\text{cosh}\;m\tau_{B}}{\text{cosh}\;m(\tau_{1}-\tau_{2})}\text{cos}\;m\theta$$ (14)

$$2\pi G_{1}^{L}({\text{x}}_{0},\text{x},n)=\sum_{m=0}^{n}\frac{4}{2m+1}\frac{\text{sin}\;(\lambda_{m}\left(\tau -\tau_{2}\right))\text{sin}\;(\lambda_{m}\left(\tau_{0}-\tau_{2}\right))}{\text{sinh}\;\lambda_{m}\pi }\text{cosh}\;\lambda_{m}\theta ,$$ (15)

and similary $G_{2}^{H}\left({\text{x}}_{0},\text{x},n\right)$ and $G_{2}^{L}\left({\text{x}}_{0},\text{x},n\right)$. Fig 3 shows how the error is distributed on the domain, when 20 terms in each series are used. Note that the error using (5) and (6), is largest close to *τ* = *τ*_0_; the error using (7) and (8) is largest close to *σ* = *σ*_0_. In practice, evaluating mean times by performing the integral (3) is most convenient using the forms (5) and (6).

## Conclusion

Green’s functions are used to calculate mean hitting or exit times of Brownian particles in confined domains whose boundaries are reflecting in some places and absorbing in others. We give exact results in two dimensions when the domain is a circle (cellular surface) with a circular inclusion (cellular nucleus). Two different types of series expression emerge when using bipolar coordinates. We sum the series to yield a closed expression involving Jacobi theta functions. The methodology of this paper can be extended to three dimensions with bispherical coordinates [27]. Transformations using bipolar and bispherical coordinates have only yielded exact results when there is a single inclusion on a circular domain. Nevertheless, exact results are useful complements to current numerical and analytical methods, accurate in certain limits, for confined diffusion with narrow exits or multiple targets [18–20, 38].

## Supporting information

S1 Code (PDF)
