# Supplementary material for: Diffusion in a disk with inclusion: Evaluating Green’s functions
Source: PLoS One. 2022 Apr 14;17(4):e0265935. doi: 10.1371/journal.pone.0265935 (PMC9009627; doi:10.1371/journal.pone.0265935)
Supplement: S1 Code — (PDF) [file pone.0265935.s001.pdf]

A Code

Here, we reproduce the code, in the language python, used to evaluate $G_1(\mathbf{x}_0, \mathbf{x})$ (12).

```python
# Stana and Lythe 2021. Exact evaluation of Green's function in 2D
# without series using Jacobi theta function.
import numpy as np
from matplotlib import cm, pyplot as plt
from mpmath import jtheta as jt, log as mlog

a = 0.1   # radius of nucleus
c = 0.25 # displacement of the nucleus
x1,x2,y1,y2 = c,0,-0.5,0 # centre of nucleus and initial position

d = 1/c*(0.25*(1+a**2-c**2)**2-a**2)**0.5
tau1,tau2 = np.log(d/a+(1+(d/a)**2)**0.5),np.log(d+(1+d**2)**0.5)

def bipolar(x,y):
    '''convert Cartesian coordinates into bipolar coordinates'''
    tau = 0.5*np.log(((x+d)**2+y**2)/((x-d)**2+y**2))
    sigma = np.pi-np.arctan2(2*d*y,x**2+y**2-d**2)
    return tau,sigma

def GBDN1(y1,y2,z1,z2):
    '''evaluate Green's function with absorbing nuclear surface and
    reflecting nuclear surface. Explicit formula using Jacobi theta functions'''
    tau,sigma = bipolar(z1+(1+d**2)**0.5, z2)
    tau0,sigma0 = bipolar(y1+(1+d**2)**0.5, y2)
    alph = np.pi/(2*(tau1-tau2))*np.complex(tau+tau0-2*tau2,np.abs(sigma-sigma0)-np.pi)
    beta = np.pi/(2*(tau1-tau2))*np.complex(tau-tau0,np.abs(sigma-sigma0)-np.pi)
    q = np.exp(-np.pi**2/(2*(tau1-tau2)))
    rat = jt(3,beta/2,q)*jt(4,alph/2,q)/(jt(4,beta/2,q)*np.abs(jt(3,alph/2,q)))
    return mlog(np.abs(rat))/np.pi

N = 100 # size of (2N+1)x(2N+1) matrix used to plot
CD = np.ones(shape=(2*N+1,2*N+1))*(-1)
z1,z2 = np.linspace(-1,1,2*N+1),np.linspace(-1,1,2*N+1)
for I in range(2*N+1):
    for J in range(2*N+1):
        CD[I,J] = GBDN1(y1,y2, z1[I], z2[J])
(Z2,Z3) = np.meshgrid(np.linspace(-1,1,2*N+1),np.linspace(-1,1,2*N+1))
HIST = CD.transpose()
HIST[np.sqrt((Z2+c)**2 + ((Z3)**2)) < a] = -100
HIST[np.sqrt((Z2**2) + (Z3**2)) > 1] = -100

fig, ax = plt.subplots(1,1, figsize=(3, 3))
plt.subplots_adjust(left=0.05, right=0.85)
myrange,z2,z3 = 0.05*np.arange(0.0,25),np.linspace(1,-1,2*N+1),np.linspace(1,-1,2*N+1)
im = ax.contourf(z2,z3,np.rot90(np.rot90(HIST)),myrange,cmap=cm.terrain)
ax.set_aspect('equal')
ax.axis('off')
ax.add_artist(plt.Circle((0,0), 1, color='blue', fill=False,linewidth=1))
ax.add_artist(plt.Circle((-c,0), a, color='red', fill=False,linewidth=1))
p0 = ax.get_position().get_points().flatten()
ax_cbar = fig.add_axes([p0[2]+p0[0], p0[1], 0.05, p0[3]-p0[1]])
cb = plt.colorbar(im, cax=ax_cbar, ticks=[0,0.6,1.2], orientation='vertical')
cb.ax.tick_params(labelsize=12)
plt.savefig('JacobiThetaG1.pdf',bbox_inches='tight')
plt.show()

# https://mpmath.org/doc/current/functions/elliptic.html#jacobi-theta-functions
```
